# Abstract of Proceedings of Medical Society of South-Western New York

**Published:** 1865-11

**Authors:** Thos. D. Strong

**Affiliations:** Sec’y pro tem.; Westfield


ART. IV.—*Abstract of Proceedings of Medical Society of South-Western New York.*

The Annual Meeting was held at Erie, November 8th. Officers elected for ensuing year:

*President*—Dr. C. K. Irwin, Dunkirk.

*Vice President*—Dr. D. D. Loop, North East.

*Secretary*—Dr. L. G. Hall, Dunkirk.

*Executive Committee*—Dr. H. W. Barrett, Dr. A. Boyd, Dr. J. H. Rathbone.

*Delegates to National Medical Society*—Dr. Wm. M. Wallace, of Erie; Dr. Thomas D. Strong, of Westfield.

Dr. Irwin, of Dunkirk, read the following paper upon the use of "Acetate of Lead in Uterine Hemorrhage, in heroic doses:"

Hemorrhage after parturition is perhaps one of the most frequent accidents of an alarming nature that the young practitioner may meet with, and no other, will cause more frequent anxious moments to the man of experience. All may have gone well with the labor, the child and placenta nicely delivered, the patient well bandaged, and the accoucher congratulate himself that he is through with the case finely, when a hurried whisper from the experienced mother or nurse reaches his ear, "Doctor she is flowing badly," starts him suddenly to his feet, and hurries him to the bed-side, with as bland and confident look as his excited nerves will permit On examination it may be that the alarm was needless, that merely a contraction of the uterus had caused an expulsion of blood in such quantity as to cause the alarm. On the other hand it may be one of those trying cases where the sanguinous fluids of the body are rapidly making their exit from this newly formed lesion, in such rapid torrents that unless speedily arrested vitality cannot be long sustained, no time is to be lost; cold is rapidly applied to the surface of the abdomen by the douche, or with napkins, also to the vialva, or perhaps the tampon is applied, the position of the patient changed, the head lowered, etc., but still the hemorrhage progresses; the symptoms are becoming more and more alarming, the patient's countenance is becoming anxious, the breathing labored and hurried, the cheeks blowing, the arms thrown wildly about, calls are made on the attendants for water and air; wants to be raised in the bed, fanned, etc.; a little stimulant is perhaps given, ammonia applied, etc. How is our poor Esculapius feeling during this trying scene of trouble? If he is of a timid nature he has probably suggested to the friends that they had better send for council, or the friends may have suggested as much to him, be that as it may, most cases you will admit will no doubt come out well with the course alluded to, with perhaps the addition of a properly adjusted pad or bandage, and a long period of confinement and rest sufficient to allow the vital fluid to again recuperate.

Cases, you know, Mr. President, do occur in which all the ordinary means I have alluded to, and many others, are employed, and still avail nothing; the patient rapidly sinks. Recently a branch of my own family suffered in the way indicated, when by the proper application of the means I am about to describe I have every confidence that a loving mother would have been spared to her household.

The remedy I propose is *acetate of lead.* "Oh, is that all? We have all used that in hemorrhage." No doubt, gentlemen, you have, but in what doses?—one, two or three grains, perhaps. Now I am about to propose to give it in doses of as many drams, and as little danger from its use as you will meet with in your two or three grain doses, and at the same time control the hemorrhage, completely, in an instant.

This treatment of cases of uterine hemorrhage, is no experiment of mine, but is the teachings of one of the best practical accouchers in this country. Dr. Joseph Workman, Professor of Obstetrics in Victoria College, taught this method to his class many years ago; but few, I believe, have ever had the courage to venture its use; yet those who have, are loud in praise of its good works.

In teaching the use of this remedy to his class, Dr. Workman did not attempt to explain its physiological action, but only its medicinal effects, which are those of a *direct emetic*, and an immediate powerful contraction of the uterus, thereby closing the mouths of the bleeding vessels. The belief of the Doctor is, that it exerts a powerful influence on the lymphatic nervous center, from which you know the uterus is so freely supplied.

You may look on this course of treatment as one which is dangerous to pursue, as the dose indicated is not warranted by the materia medica of the present day, but pronounced poisonous and dangerous.

A few years ago olium terebinth, iodide potassium, quinine, and a host of other remedies were laid down in the books as unsafe, in quantities such as are now freely and safely given every day. Can any of you describe the physiological effect of cinchonia or quinine in intermittent fever? Probably not, and yet I doubt if any of you would object to their use in such cases; neither would you hesitate to give an ounce of oleum terebinth to expel the linea solium, yet we owe the credit of this last experiment to the ignorant sailor who risked his life in this way to relieve himself of the miseries caused by a tape worm.

Having always used the acetate of lead in my practice in doses of from one to three drams, without having lost a patient by hemorrhage from the uterus, I feel it my duty to explain and defend its use, believing that a remedy of such utility should not be confined to the practice of a few only. It is not necessary to confine its use to cases of full period, or where the placenta has been delivered, as its action will be to cause immediate expulsion of the contents of the uterus, and I would use it, in case of violent hemorrhage from polypus, hydatids, abortions, retention of placenta, or almost any case requiring prompt and heroic action for the suppression of uterine hemorrhage, except in cases of placenta previa.

I therefore, recommend to my colleagues the use of this remedy in cases of uterine hemorrhage from whatever cause, (save placenta previa,) where prompt action and potent remedies are indicated. I am satisfied its administration is as free from danger as that of a dose of ergot tea. The most convenient mode of administration is by putting a teaspoonful or more of the crystals of acetate of lead in a cup of plain tea, and adding thereto a tablespoonful of vinegar to insure against its being a diacetate or carbonate, either of which might not answer the purpose of the acetate. A dose of ipecac might safely be held in readiness in case vomiting was not speedily produced by the lead, but I have never had to resort to such means, as the lead has always acted, most promptly.

Dr. Wallace had been in the habit of using scruple dos with good effect, not producing emesis.

Dr. Strong had tried Dr. Irwin's treatment in one case of abortion at three months, with retained placenta and active hemorrhage. A teaspoonful of lead was given in tea, with a little vinegar added. The taking of the lead, emesis and the expulsion of the placenta were almost instantaneous, thus terminating the flowing.

Neither Dr. Irwin or Dr. Wallace had seen any ill effects from the lead.

At request of Dr. Wallace, Dr. Bennett reported his experience in the use of Fowler's Solution in typho-malarial fever. Dr. Bennett was accustomed to use freely in the army, ten drops three times daily; did not hesitate to use it if stomach did not rebel, or puffiness appear under the eyes. Was much pleased with its effect.

Drs. Loop and Irwin coincided with him in regarding it as a valuable agent. Dr. Irwin said that he had frequently obtained satisfactory results from it, in intermittents, when quinine had failed.

Dr. Hazeltine reported a great prevalence of icterus, not attended with fever, in south part of the county.

Dr. Loop reports the same at North East. Did not require much treatment. Thos. D. Strong, *Sec'y pro tem.*

Westfield, Nov. 13, 1865.

